# Lu’s approach for video-assisted thoracoscopic surgery

**DOI:** 10.1371/journal.pone.0300632

**Published:** 2024-06-25

**Authors:** Baofeng Wang, Jiang Wang, Tongyu Sun, Yilin Ding, Shasha Li, Hengxiao Lu

**Affiliations:** 1 School of Clinical Medicine, Shandong Second Medical University, Weifang, China; 2 Department of Thoracic Surgery, Weifang people’s Hospital, Weifang, China; 3 Clinic, Weifang People’s Hospital, Weifang, China; European Institute of Oncology: Istituto Europeo di Oncologia, ITALY

## Abstract

**Objectives:**

Lu’s approach for video-assisted thoracoscopic surgery (LVATS), which derives from UVATS, is a novel surgical approach for VATS and carries out micro-innovation for lung cancer resection. The objective of this study is to elucidate the safety, feasibility, and efficacy of this novel surgical approach.

**Methods:**

The clinical data of patients with non-small cell lung cancer (NSCLC) who underwent a curative thoracoscopic lobectomy between Mar. 2021 and Mar. 2022, were retrospectively collected, and analyzed. According to whether applied Lu’s approach during the VATS operation, patients were divided into the LVATS group and the UVATS group. The propensity score (PS) matching method was used to reduce selection bias by creating two groups. After generating the PSs, 1:1 ratio and nearest-neighbor score matching was completed. Perioperative variables, including the operation time, intraoperative blood loss, lymph node stations dissected, total drainage volume, drainage duration, postoperative hospital stay, pain score (VAS, Visual Analogue Scale) on the postoperative first day (POD1) and third day (POD3), and incidence of postoperative complications, were compared between the two groups. The data were analyzed statistically with P<0.05 defined as statistically significant.

**Results:**

A total of 182 patients were identified, among whom 86 patients underwent LVATS and 96 UVATS. Propensity matching produced 62 pairs in this retrospective study. There were no deaths during perioperative period. Patients in the LVATS group experienced a shorter operation time (88 (75, 106) VS 122 (97, 144)min, P <0.001), less intraoperative blood loss(20 (20, 30) VS 25 (20, 50)ml, P = 0.021), shorten incision length (2.50 (2.50, 2.50) VS 3.00 (3.00, 3.50)cm, P <0.001), and more drainage volume (460 (310, 660) VS 345 (225, 600)ml, P = 0.041) than patients in the UVATS group. There was not significant difference in the lymph node stations dissected(5 (4, 5) VS 5 (4, 5), P = 0.436), drainage duration (3 (3, 4) VS 3 (3, 4)days, P = 0.743), length of postoperative hospital stay (4 (4, 5) VS 4 (4, 6)days, P = 0.608), VAS on the POD1(4 (4, 4) VS 4 (4, 4), P = 0.058)and POD3 (3 (3, 4) VS 4 (3, 4), P = 0.219), and incidence of postoperative complications (P = 0.521) between the two groups.

**Conclusions:**

Lu’s approach is a safe and feasible approach for video-assisted thoracoscopic surgery for the lobectomy of NSCLC. This approach can shorten surgical time, reduce incision length and intraoperative blood loss.

## Introduction

Lung cancer is the leading cause of cancer-related mortality worldwide. Non-small cell lung cancer (NSCLC) is the most common type, accounting for approximately 85% of all lung cancer patients [1]. Currently, surgical resection is the primary mode of treatment for early-stage NSCLC [2]. In recent years, video-assisted thoracic surgery (VATS) and enhanced recovery after surgery have greatly improved the safety, tolerance, and efficacy of lobectomies for early-stage NSCLC patients. Uniportal VATS (UVATS) has been reported to be more feasible than multiportal VATS, resulting in less blood loss, shorter postoperative drainage times, and other benefits [3]. However, some opponents of UVATS have pointed out technical challenges with this approach. Due to the crowding of the scope and instruments, dexterity can be compromised, and it has been compared to a game of Twister for the surgeon and assistant [4]. Based on the micro-innovation of UVATS, we have proposed the Lu’s approach for video-assisted thoracoscopic surgery (LVATS) for early NSCLC patients. Currently, this novel surgical approach has not been reported internationally.

The aim of this study is to introduce our technical approach and implementation process and to clarify the safety, feasibility, and efficacy of LVATS for the treatment of patients with early NSCLC.

## Materials and methods

The study was approved by the institutional review board and the ethics committee of Weifang People’s Hospital. Preoperative examination including electrocardiography, pulmonary function test, chest computed tomography scan, bronchoscope and brain magnetic resonance imaging. Date of access to data for study purposes was 06 September 2023. The medical records of 182 patients who underwent thoracoscopic lobectomy in the Thoracic Surgery Department of Weifang People’s Hospital from March 2021 to March 2022 were retrospectively collected and analyzed. Patients were assigned to the LVATS group (n = 86) and the UVATS group (n = 96), based on whether the Lu’s approach are applied during the operation. All of the procedures were performed by one thoracic surgical team that used the same clinical protocols, care patterns, and perioperative orders. And they were performed by the same first surgeon who has completed his UVATS learning curve and is proficient in UVATS.

The inclusion criteria were:

1. NSCLC patients with primary and single lung lesions;
2. with no previous thoracic interventions or surgery on the affected side;
3. clinical staging of T1–T3 and N0–N2, and no distant metastases;
4. patients with an American Society of Anesthesiologists (ASA) score of I–II.

The exclusion criteria included:

1. sub-pulmonary lobectomy and preoperative neoadjuvant therapy;
2. cT3 tumors invading to the chest wall, diaphragm or pericardium;
3. had a history of malignancies other than NSCLC;
4. patients with history of chest surgery.

Clinical features including age, sex, smoking history, hypertension, diabetes, tumour location and diameter, tumor stage, histology, operation duration, estimated volume of blood loss, lymph node stations dissected, total drainage volume, drainage duration, postoperative hospital stay, VAS on the POD1 and POD3, and postoperative complications were recorded. The operation duration is the primary perioperative outcomes and the others are secondary.

### Surgical technique of VATS lobectomy

#### Anaesthesia and analgesia

All patients received a general anaesthesia, and were provided with patient-controlled analgesia postoperatively. After intravenous induction, each patient was intubated with a double-lumen endotracheal tube to accomplish single-lung ventilation. Patients’ vital signs were followed during the operation. All patients were extubated at the end of surgery and transferred to the ward.

#### Position

**Fig 1:**
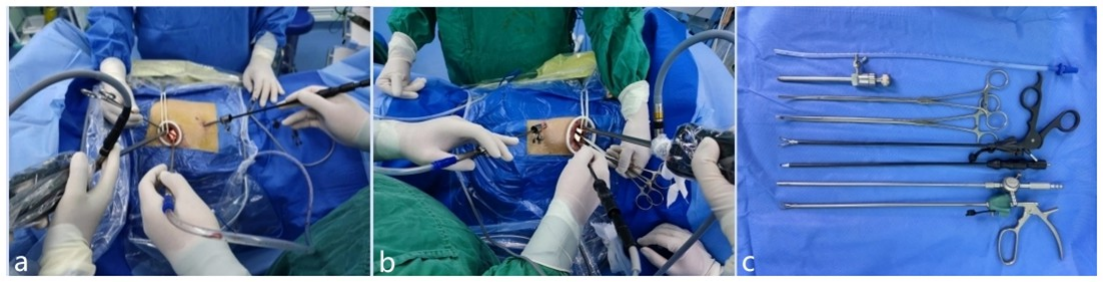
Surgical set up for Lu’s approach for video-assisted thoracoscopic surgery (LVATS). **a** The right-handed energy instrument approach in left-sided lung surgery, the electric hook was introduced by a 5 mm trocar hole that was used as a drainage port after surgery, and the surgical instruments such as aspirator was introduced by the main operation port. The surgeon and camera holder were positioned on the ventral side and the assistant was positioned on the dorsal side of the patient. **b** In the right-sided lung surgery, the energy instrument in the right hand was introduced by the main operation port and the surgical instrument with a diameter of less than 5 mm such as aspirator was introduced by the 5 mm trocar hole. The intraoperative position of the surgeon was the same as for left-sided lung surgery. **c** The surgical instruments in LVATS.

The patient was kept in a folding knife gesture in the lateral decubitus position. Intraoperatively, the surgeon and the camera holder stood on the abdominal side of the patient, the assistant stood on the back side of the patient in the LVATS group (Fig 1a and 1b). The technical details of the classic UVATS have been described in detail in previous publications [5].

#### Video-assisted thoracoscopic surgery lobectomy

In LVATS lobectomy, a 2.0 to 3.0-cm incision was made at the fourth intercostal space along the anterior axillary line for any lobe resection. A soft plastic wound protector was applied to the incision without rib-spreading. The 10-mm 30° thoracoscope was introduced in the superior side of the incision during the operation. Different from UVATS, a 5mm incision was made at the seventh intercostal space along the midaxillary line and a 5mm trocar was placed in this incision to establish a channel (Fig 1a and 1b), through which any instrument with a diameter less than 5mm, such as energy instrument, aspirator, separation forceps and vascular clamp (Fig 1c), could be introduced to avoid instrument collision caused by single-port operation (Fig 1). The next procedures were as described in a previous publication [5]. The adhesions were separated, inferior pulmonary ligament was dissociated, and the pleura of pulmonary hilar was opened, the bronchus, vein and artery were divided anatomically, then cut and close the pulmonary veins, pulmonary arteries and bronchi with a laparoscopic cutting closer. Subsequently, a standard lymphadenectomy was carried out. All specimens were placed in an endoscopic plastic bag under thoracoscopic assistance and removed through the operational incision. Finally, the chest was rinsed using normal saline, and the bleeding was examined. Single lung ventilation was changed to double lung ventilation, and expansion of lung lobe and leakage were checked. A 16 Fr chest tube was inserted through the 5mm port to achieve low position thoracic drainage at the end of the operation, and this would be removed in case there was no air leakage and the volume of drainage was less than 200 ml per day postoperatively.

In UVATS lobectomy, a 3.0 to 4.0-cm incision was made at the fourth intercostal space along the anterior axillary line for upper lobe resection and the fifth intercostal space for middle and inferior lobe resection. In most cases, the thoracoscope is placed on the posterior side of the incision and the other working instruments are placed on the anterior side of the incision. The next procedure is roughly same as the LVATS group, the bronchus, vein and artery were divided anatomically, then cut and close the pulmonary veins, pulmonary arteries and bronchi with a laparoscopic cutting closer. The specimen was taken out in the specimen bag. Conventionally, systemic mediastinal lymph node dissection would be performed after the removal of the target lobe. After pulmonary lobectomy and lymphadenectomy, a 16 Fr chest tube was inserted through the incision, and this would be removed in case there was no air leakage and the volume of drainage was less than 200 ml per day postoperatively.

### Statistical analysis

R version 4.2.1 was used for statistical analysis. Propensity score-matched analysis was performed for the comparison between LVATS and UVATS lobectomies. The two pairs were matched according to the propensity score. The included parameters were age, sex, tumour histology, tumour diameter, the completeness of the fissure and the extent of pleural adhesion. The one–to–one match was achieved by using the nearest neighbour-matching algorithm. Continuous variables were presented as mean and interquartile spacing, and Wilcoxon rank sum test or t test was used for comparison between groups. The Chi-square test or Fisher’s exact test was used for categorical variables, and rank sum test for quantitative variables. P < 0.05 was considered statistically significant.

## Results

### Clinical features

A total of 182 patients were identified, and divided into the two groups: the LVATS (n = 86) and the UVATS (n = 96). 62 LVATS lobectomies and 62 UVATS lobectomies were matched after the propensity scored analysis in this retrospective study. The clinical features of the study cohort before and after matching are listed in Table 1. There was no major difference between the two groups in the demographic information: age (P = 0.828), gender (P = 0.718), smoking history (P>0.999), hypertension(P = 0.326), diabetes (P = 0.769), COPD (P>0.999), tumor location (P = 0.994), tumor diameter(P = 0.251).

**Table 1:** Clinical features before and after matching.

	All patients			Propensity-matched patients		
	UVATS	LVATS	p-value^2^	UVATS	LVATS	p-value^2^
	(n = 96^1^)	(n = 86^1^)		(n = 62^1^)	(n = 62^1^)	
**Gender**			0.101			0.718
Female	51 (53%)	56 (65%)		35 (56%)	33 (53%)	
Male	45 (47%)	30 (35%)		27 (44%)	29 (47%)	
**Age**	58 (51, 65)	58 (52, 64)	0.800	57 (51, 63)	58 (52, 63)	0.828
**Smoking**			0.298			>0.999
No	72 (75%)	70 (81%)		47 (76%)	47 (76%)	
Yes	24 (25%)	16 (19%)		15 (24%)	15 (24%)	
**Hypertension**			0.186			0.326
No	64 (67%)	65 (76%)		41 (66%)	46 (74%)	
Yes	32 (33%)	21 (24%)		21 (34%)	16 (26%)	
**Diabetes**			0.620			0.769
No	87 (91%)	76 (88%)		55 (89%)	56 (90%)	
Yes	9 (9.4%)	10 (12%)		7 (11%)	6 (9.7%)	
**COPD**			0.275			>0.999
No	82 (85%)	78 (91%)		54 (87%)	54 (87%)	
Yes	14(15%)	8(9.3%)		8 (13%)	8 (13%)	
**Tumor location**			0.081			0.994
LLL	8 (8.3%)	15 (17%)		6 (9.7%)	6 (9.7%)	
LUL	25 (26%)	24(28%)		19(31%)	18 (29%)	
RLL	19 (20%)	14 (16%)		11 (18%)	13 (21%)	
RML	10 (10%)	15 (17%)		7(11%)	7(11%)	
RUL	34 (35%)	18 (21%)		19 (31%)	18 (29%)	
**Diameter**	1.20 (0.80, 1.60)	1.30 (0.90, 1.80)	0.561	1.25 (0.80, 1.58)	1.40 (1.00, 1.80)	0.251

^1^n (%); Median (IQR)

^2^Pearson’s Chi-squared test; Wilcoxon rank sum test.

Note:1, left lower lobe. 2, left upper lobe. 3, right lower lobe. 4, right middle lobe. 5, right upper lobe

The primary clinical result is the operation duration. The operation duration in LVATS was shorter than in UVATS (88 (75, 106) VS 122 (97, 144) min, P <0.001). The volume of estimated blood loss in LVATS was less than in UVATS (20 (20, 30) VS 25 (20, 50)ml, P = 0.021). The LVATS had shorten incision length (2.50 (2.50, 2.50) VS 3.00 (3.00, 3.50) cm, P <0.001) than UVATS. Patients in LVATS group had more drainage volume (460 (310, 660) VS 345 (225, 600) ml, P = 0.041) than in UVATS group. While the lymph node stations dissected (5 (4, 5) VS 5 (4, 5), P = 0.436), and drainage duration (3 (3, 4) VS 3 (3, 4), P = 0.743)days, length of postoperative hospital stay (4 (4, 5) VS 4 (4, 6), P = 0.608)days, VAS on the POD1(4 (4, 4) VS 4 (4, 4), P = 0.058) and POD3(3 (3, 4) VS 4 (3, 4), P = 0.219)were similar between the two groups. And the postoperative complications between two groups were similar (P = 0.521). The clinical results are recorded in Table 2.

**Table 2:** Comparison on perioperative outcomes.

Variable	UVATS, N = 62^1^	LVATS, N = 62^1^	p-value^2^
**time (min)**	122 (97, 144)	88 (75, 106)	<0.001
**bleeding (mL)**	25 (20, 50)	20 (20, 30)	0.021
**lymph node dissection Stations (station)**	5 (4, 5)	5 (4, 5)	0.463
**chest tube indwelling time (day)**	3 (3, 4)	3 (3, 4)	0.743
**drainage volume (mL)**	345 (225, 600)	460 (310, 660)	0.041
**incision (cm)**	3.00 (3.00, 3.50)	2.50 (2.50, 2.50)	<0.001
**postoperative hospital stay (day)**	4 (4, 6)	4 (4, 5)	0.608
**POD1 VAS (point)**	4 (4, 4)	4 (4, 4)	0.058
**POD3 VAS (point)**	4 (3, 4)	3 (3, 4)	0.219
**Postoperative complication**			0.521
bacterial pneumonia	0 (0%)	3 (4.8%)	
chylothorax	1 (1.6%)	1 (1.6%)	
N	59 (95%)	55 (89%)	
postoperative leakage	1 (1.6%)	2 (3.2%)	
pulmonary atelectasis	1 (1.6%)	1 (1.6%)	

^1^Median (IQR); n (%)

^2^Wilcoxon rank sum test; Fisher’s exact test

### Morbidity and mortality

No mortality was recorded in either group. A total of 16 patients developed complications (9 vs 7 in LVATS and UVATS, respectively), including 4 prolonged air leakages lasting for more than 5 days, 3 atelectasis and 7 bacterial pneumonias and 2 chylothoraxs requiring prolonged drainage. All patients were discharged following the confirmation of lung re-expansion by chest X-rays or CT. The average postoperative length of stay in hospital was 5.38±3.06 days in LVATS and 5.66±4.95 days (P = 0.608) in UVATS. Prolonged stay or readmission to the intensive care unit was not recorded in this cohort.

## Discussion

**Fig 2:**
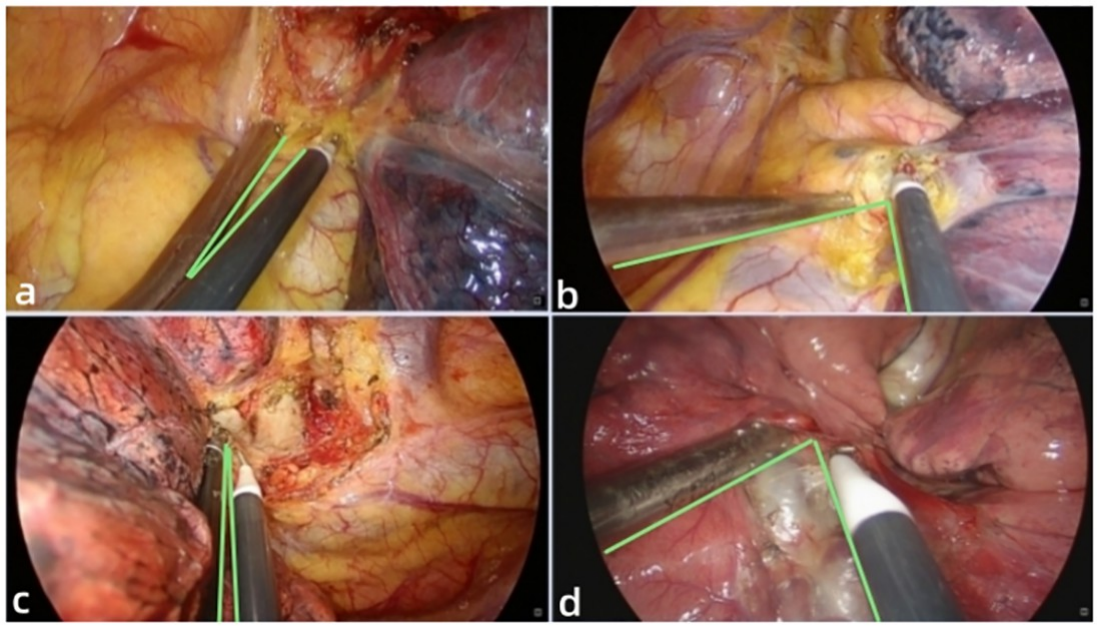
The contrast between the angles formed by two surgical instruments in the UVATS and LVATS, maintaining a large angle between the two instruments can reduce collisions between instruments and facilitate the surgeon ’s operation. **a** the angle between the electric hook and the aspirator during the dissection of the left hilus in UVATS. **b** the angle between the electric hook and the aspirator during the dissection of the left hilus in LVATS. **c** the angle between the electric hook and the aspirator during the dissection of the right hilus in UVATS. **d** the angle between these two instruments during the dissection of the righ lung fissure in LVATS.

**Fig 3:**
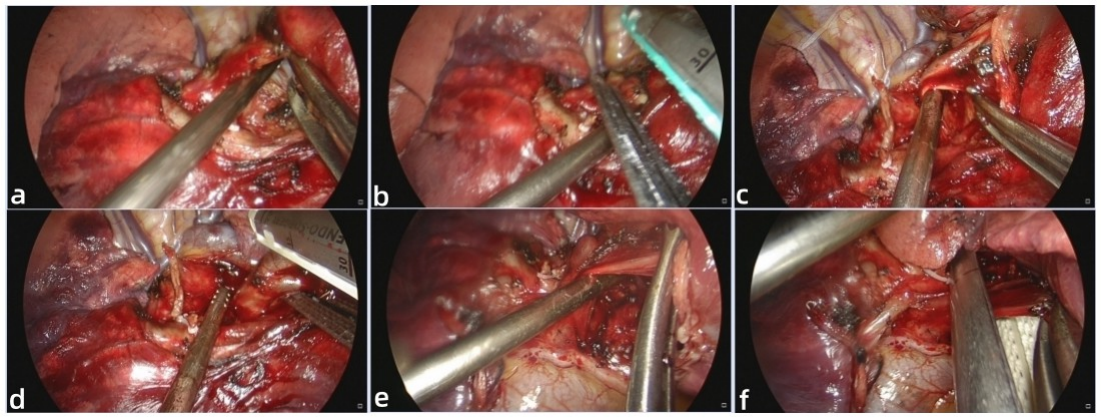
LVATS can bring convenience to the placement of cutting closure device during surgery. **a** the aspirator introduced through the drainage port can assist the separation of bronchus of the right upper lobe in LVATS. **b** the aspirator introduced through the drainage port can assist placing the cutting closure device to cut and close the bronchi of the right upper lobe in LVATS. **c** the aspirator assisted separating the artery of the right upper lobe in LVATS. **d** the aspirator assisted placing the cutting closure device to cut and close the artery of the right upper lobe in LVATS. **e** the aspirator assisted separating the vein of the right upper lobe in LVATS. **f** the aspirator assisted placing the cutting closure device to cut and close the vein of the right upper lobe in LVATS.

Lung cancer is a major global health concern, with high morbidity and mortality rates. It is broadly categorised into two types: small cell lung cancer and non-small cell lung cancer (NSCLC) [6]. NSCLC, accounting for approximately 85% of all lung cancer patients, has several main subtypes, including adenocarcinoma, squamous cell carcinoma, undifferentiated carcinoma, sarcomatoid carcinoma, and adenosquamous carcinoma [7]. Several risk factors have been identified for NSCLC, such as history of smoking, asbestos exposure, exposure to metal and mineral dust, and air pollution [8]. Currently, surgical resection remains the primary mode of treatment for early-stage NSCLC and is an essential component of multimodality therapy for even more advanced disease with curative intent [9]. In this retrospective study, we compared LVATS and UVATS lobectomies for non-small cell lung cancer (NSCLC). We found that LVATS was safe and feasible for the surgical resection of lung cancer and had a shorter duration than traditional UVATS lobectomy. Our results from a single center showed high efficacy for LVATS. In 2011, Gonzalez-Rivas reported the first case of UVATS lobectomy [10]. UVATS represents a further development of classic VATS under the concept of minimally invasive and rapid recovery, with advantages such as reduced incision size, improved field of view, and reduced chest wall and intercostal nerve injuries compared to multi-port VATS [11]. However, UVATS also presents challenges due to restrictions on the instruments used, including the thoracoscope [12]. The major challenges of UVATS include limited tool usage, regulation of tool insertion directions, interference between tools, difficulty obtaining a clear view due to collinear view and tool axes, bleeding control, and suturing [13]. All operating tools and the thoracoscope are placed in the same port in UVATS, resulting in a narrow operating space and making cooperation between the surgeon and assistant difficult. Interference between instruments and the mirror body can also lead to an extended learning curve and increased surgical difficulty. Thus, clinicians must invest significant effort in learning UVATS and fully mastering the procedure, with a lot of surgical practice being essential [14]. Despite the benefits of UVATS, concerns over operative risks, technical challenges, and unstudied outcomes remain for UVATS lobectomy [15]. As such, there is a need to ensure effective thoracoscopic surgery while reducing surgical difficulty, improving operative safety, shortening operation time, and decreasing the learning curve. Our proposed LVATS builds on the classic UVATS approach. In comparing postoperative results between the two groups, the primary outcome was that the LVATS group showed significantly lower intraoperative blood loss than the UVATS group, the operation duration for LVATS was shorter than traditional UVATS. While lacking objective evidence, LVATS has contributed to improved ergonomics and comfort level of the surgeon throughout the whole procedure. The angle between the two instruments in LVATS is bigger than that of UVATS, which can effectively avoid collisions between the two instruments during surgery, make surgeon more convenient, comfortable and safe to perform operations such as exposure, dissection and hemostasis and reduce operation time (Fig 2). The limited instrumentation and difficult angles for vessel dissection and stapling with instruments pose significant challenges for the UVATS surgeon [16]. The aspirator introduced through the Lu’s approach can more effectively assist the surgeon in separating and exposing the bronchus, arteriovenous vessels and provide the surgeon with a better angle for the placement of the cutting closure device in LVATS (Fig 3). Additionally, the single small incision and limited intercostal space can cause intense jamming and interference between the thoracoscope and instruments, leading to ergonomic discomfort for surgeons and longer operation times for patients undergoing UVATS [17]. In LVATS, the 5mm trocar was placed in the seventh intercostal space along the midaxillary line for LVATS, allowing for the introduction of any instrument less than 5mm in diameter (energy instruments, aspirator, separation forceps, vascular clips, e.g.), thus, reduced the degree of intraoperative space limitation and avoided the operation inconveniences caused by cross-collision between instruments and coaxial operation. Therefore, the LVATS is able to overcome some of the limitations of UVATS, such as mutual interference between instruments and insufficient exposure [18], reducing the difficulty and surgical risk in UVATS. Intraoperative bleeding is a significant safety concern associated with VATS for a lobectomy. The major causes of blood loss during the operation were pleural atresia or dense adhesions of the pleural cavity, vascular injury, pulmonary parenchymal hemorrhage, and chest wall oozing [19]. UVATS is more challenging than traditional multi-port thoracoscopic surgery and cannot effectively deal with severe adhesion diseases and intraoperative bleeding [20]. As for the resection of the larger NSCLC, it has been shown that UVATS is safe and effective for NSCLC larger than 5 cm, but conversion to multiportal VATS or even open surgery may be necessary when there is a risk of uncontrollable intraoperative bleeding or incomplete resection of the tumor [21]. The efficacy of LVATS might be comparable to UVATS in resecting larger NSCLC although there is no study have demonstrated. In these special cases, surgeons are able to respond more easily and effectively by applying Lu’s approach in surgery and some intraoperative operations are no longer challenging for surgeon in LVATS.

**Fig 4:**
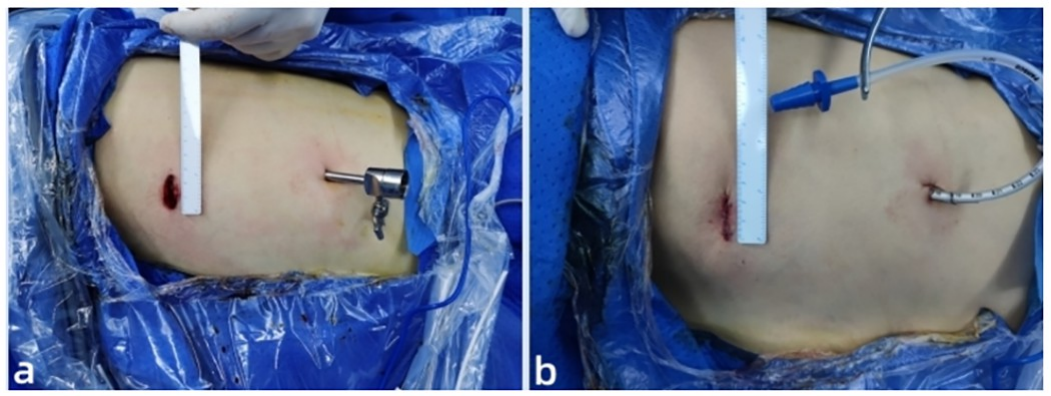
Appearance of the LVATS incisions. **a** details of surgical incision and the 5 mm trocar was introduced in LVAST. **b** the 5mm drain port achieved low thoracic drainage postoperatively in LVATS.

Furthermore the LVATS provides a similar surgical perspective to UVATS and thoracotomy, which effectively relieves visual fatigue and facilitates direct vision operation [22]. As for the length of the incision, the LVATS group was significantly smaller than that of UVATS group. In LVATS, by applying the Lu’s approach, the drainage tube port can be exploited as an auxiliary operation port, the 3-4cm incision in UVATS can be reduced to approximately 2.5cm without affecting the operation (Fig 4).

The postoperative drainage volume of LVATS group was more than that of UVATS group. This may suggest that LVATS can achieve better postoperative drainage than UVATS. In UVATS, a chest tube is placed through the incision to drain the effusion and gas from the thoracic cavity after the operation. This often results in poor drainage of pleural effusion. And also, the skin around the tube is not well aligned, easily leading to poor incision healing and gas and effusion leakage [23]. In LVATS, the seventh intercostal port was applied in advance as an auxiliary operation port intraoperatively, and a 16Fr chest tube was introduced as a drainage port postoperatively to achieve low position drainage (Fig 4).

This study has certain limitations, including a small sample size, a lack of accurate quantitative indicators, the potential influence of operators’ skills and proficiency on certain surgical and postoperative observation indicators, and a lack of long-term efficacy comparison. Therefore, a more scientifically based plan should be formulated to enhance the quality of research outcomes in the next stage. This will help to improve our understanding of the topic and ultimately result in more meaningful conclusions.

## Conclusion

In summary, Lu’s approach for video-assisted thoracoscopic surgery (LVATS) lobectomy is a safe, feasible, and effective procedure for treating patients with early NSCLC. This technique has the potential to reduce operation duration, incision length and intraoperative blood loss and achieve better drainage of postoperative pleural effusion, which can ultimately benefit both patients and medical professionals.

## Supporting information

S1 Data (CSV)

## Abbreviations

LVATS: Lu’s approach for video-assisted thoracoscopic surgery
NSCLC: non-small cell lung cancer
POD: postoperative day
UVATS: video-assisted thoracoscopic surgery
VAS: Visual Analogue Scale
